# Comparison of long-term complications in cancer patients with incidental and acute symptomatic venous thromboembolism

**DOI:** 10.3389/fcvm.2023.1118385

**Published:** 2023-05-19

**Authors:** María Barca-Hernando, Sergio Lopez-Ruz, Samira Marin-Romero, Teresa Elias-Hernandez, Remedios Otero-Candelera, Luis Jara-Palomares

**Affiliations:** ^1^ Respiratory Department, Medical Surgical Unit of Respiratory Diseases, Hospital Virgen del Rocio, Sevilla, Spain; ^2^ Centro de Investigación Biomédica en Red de Enfermedades Respiratorias, Instituto de Salud Carlos III, Madrid, Spain

**Keywords:** neoplasms, incidental, venous thromboembolism, venous thrombosis, hemorrhage, mortality

## Abstract

**Background:**

Clinical practice guidelines recommend that patients with incidental venous thromboembolism (VTE) receive the same anticoagulant therapy as those with symptomatic VTE. We aimed to compare the rate of complications between cancer patients with incidental and symptomatic VTE through a long-term follow-up cohort.

**Methods:**

We performed a *post hoc* analysis of prospective studies of cancer patients with VTE between 2008 and 2019, with the primary outcome of rates of recurrent VTE and clinically relevant bleeding (CRB) in incidental and symptomatic VTE groups.

**Results:**

In total, 796 patients were included, of which 42.8% had incidental VTE. No significant differences were noted in the rate of recurrent VTE (0.4 per 100 patients/month vs. 0.5 per 100 patients/month; *p* = 0.313) and in the rate of CRB (0.6 per 100 patients/month vs. 0.5 per 100 patients/month; *p* = 0.128) between patients with incidental VTE and symptomatic VTE, respectively. At six-month follow-ups, the cumulative incidence of CRB was significantly higher in patients with incidental VTE than that in those with symptomatic VTE (7.9% vs. 4.4%, respectively; OR: 1.8; 95% CI: 1.01–3.2).

**Conclusion:**

Cancer patients with incidental VTE had similar rates of CRB and VTE recurrence in long-term follow-up compared with patients with symptomatic VTE. At six-month follow-ups, patients with incidental VTE had a higher cumulative incidence of CRB than those with symptomatic VTE.

## 1. Introduction

Venous thromboembolism (VTE), a condition that includes pulmonary embolism (PE) and deep vein thrombosis (DVT), is a frequent complication of cancer (1, 2). These patients have a 6 to 14-fold higher risk of developing VTE than patients without cancer (1, 2), and a higher incidence of recurrent VTE and hemorrhages (3, 4). The pathogenesis of VTE in cancer patients is multifactorial, and involves several pathways: direct coagulation pathway activation, induction of inflammatory responses, inhibition of fibrinolytic activity, and tumor cell-induced platelet aggregation (5). Incidental VTE is commonly diagnosed in those undergoing routine computed tomography scans at initial staging periods of oncologic disease or in the evaluation of oncologic treatments (6). In cancer patients, the rate of incidental diagnosis of PE varies from 3.1% to 9% (7–10), and approximately 50% of PE cases are incidentally diagnosed (11).

Clinical practice guidelines recommend the same anticoagulant therapy for a minimum of 3–6 months in cases of both incidental and symptomatic VTE (12–15). However, current data on incidental VTE are generally based on limited retrospective studies. One international prospective study found 12-month cumulative incidences of recurrent VTE and major bleeding of 6.0% (95% CI: 4.4%–8.1%) and 5.7% (95% CI: 4.1%–7.7%), respectively (8, 16, 17). Additionally, a recent systematic review and meta-analysis that compared outcomes among patients with cancer and incidental or symptomatic VTE was conducted, but only three randomized controlled trials were included in the final analysis due to observational studies that were too heterogeneous and of medium to low quality (18). Moreover, subgroup analyses in randomized controlled trials may have included under-or overestimated subgroup effects (19). Therefore, more data regarding the long-term complications of incidental vs. symptomatic VTE, such as recurrence, bleeding, and death, are needed. We aimed to compare the rates of complications in cancer patients with incidental or symptomatic VTE in a long-term follow-up cohort.

## 2. Materials and methods

We performed a post-hoc analysis of prospective studies containing consecutive patients with cancer and VTE [2013PI/200, Hispalis (20); TiCAT (21); 0191-N-14: Qca (22)]. This study was evaluated and approved by the appropriate ethics committee of the center, according to Spanish regulatory authorities (0511-N-22), and conducted according to the principles of the Declaration of Helsinki and ICH Guidelines for Good Clinical Practice, in full conformity with relevant regulations. All study documents were prepared in accordance with Good Clinical Practice guidelines (CPMP/ICH/135/95). Data were obtained for this project, anonymized, and protected according to the European Union Directive 2016/679 of the European Parliament and the European Council on April 27, 2016.

### 2.1. Patient selection

Cancer patients with a confirmed diagnosis of incidental or acute symptomatic VTE (DVT, PE, or unusual site VTE) were consecutively recruited at the Hospital Universitario Virgen del Rocio de Sevilla (Spain) from October 2008 to December 2019. Follow-ups of all patients were conducted through September 2021. Incidental VTE was defined as a diagnosis made based on radiological imaging tests performed for reasons other than clinical suspicion of VTE. We defined unusual site VTE as thrombosis that affects any venous region other than pulmonary thromboembolism or deep vein thrombosis of limbs (lower or upper). Complications, such as recurrent VTE, clinically relevant bleeding (CRB), and death, were recorded by two investigators (MBH and SLR) and independently adjudicated (SMR and LJP). Confirmation of the VTE using computed tomography, pulmonary scintigraphy, or compression ultrasound was required for all patients (23, 24), and tumor diagnoses were confirmed through histological exams. Additionally, patients who could not be followed-up or were aged <18 years were excluded.

### 2.2. Primary and secondary outcomes

The primary outcome was the rate of recurrent VTE and CRB during follow-up in two cohorts of patients: cancer patients with incidental VTE or acute symptomatic VTE. Secondary outcomes included (1) cumulative incidence of recurrent VTE, CRB, major bleeding, and mortality at six and 12 months; (2) rate of major bleeding under anticoagulant treatment during follow-up; (3) all-cause mortality; and 4) subgroup analyses, including oncological treatment (yes/no), sex (male/female), age (≤ 65 vs. >65 years), metastases (yes/no), and Eastern Cooperative Oncology Group (ECOG) performance status (0 vs. >0). Recurrent VTE was defined as objectively confirmed symptomatic or incidental VTE by imaging with evidence of thrombus progression or involvement of the thrombus in another region. Major bleeding was evaluated according to the following criteria of the International Society on Thrombosis and Hemostasis (ISTH): fatal bleeding; bleeding in a critical area or organ, such as intracranial, intraspinal, intraocular, retroperitoneal, intraarticular, pericardial, or intramuscular; organs with compartment syndrome; and/or hemorrhage producing a reduction in hemoglobin levels ≥20 g/L or requiring transfusion with >2 units of total blood or red blood cells (25). CRB includes major bleeding and clinically relevant non-major bleeding defined, according to the ISTH criteria, as any bleeding requiring professional medical intervention, hospital admission, or in-person evaluation (25).

### 2.3. Statistical analyses

Normally distributed quantitative variables were expressed as mean ± standard deviation or median and percentile 25–75 (p25–p75), whereas qualitative variables were given as numbers and proportions. Categorical variables were compared using chi-squared or Fisher's exact tests, while continuous variables were compared using Student's *t*-tests or Mann–Whitney *U*-tests. The rate was defined as the number of patients with an event (CRB or recurrent VTE) divided by the total number of patients and the month at risk of the event. To estimate the time to event, such as bleeding, VTE recurrence, or death, Kaplan–Meier probabilities were computed, and differences between the groups were assessed using the log-rank test. Survival analyses were censored for loss to follow-up, end of study, event of interest, or death. Cox regression with non-parametric adjustment of proportional risks was performed based on oncological treatment (yes/no), sex (male/female), age (≤65 vs. >65 years), metastases (yes/no), and ECOG performance status (0 vs. >0). Hazard ratios (HR) and 95% confidence intervals (CI) were calculated, and adjusted HR (aHR) were obtained after multivariate analysis. The IBM SPSS Statistics program (version 21; SPSS Inc., Chicago, IL, USA) was used for all statistical analyses, and *p* values of <0.05 were considered statistically significant.

## 3. Results

**Figure 1:**
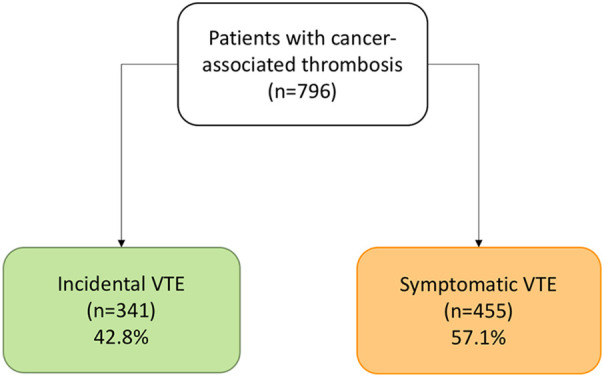
Flow diagram.

In total, 796 patients with cancer and VTE were included from October 2008 to December 2019, of which 341 patients (42.8%) had incidental VTE and 455 had symptomatic VTE (57.2%) (Figure 1). The mean age was 64.3 ± 13.3 years, 55.5% of the patients had metastases, and 70.9% were undergoing cancer treatment at the time of VTE diagnosis. In addition, the majority of patients (57%) presented with PE, with or without associated DVT. The median duration of anticoagulant therapy was 9.5 months (p25–p75, 5.4–19.4), and the most common anticoagulant treatment was low-molecular-weight heparin (LMWH) (97.7%). Furthermore, the median follow-up time was 20.4 months (p25–p75, 7.5–40.2), with a rate of 1.972 patients/year. The clinical characteristics of the patients are summarized in Table 1.

**Table 1:** Baseline characteristics of patients.

Variable	*n* (*n* = 796)
Male sex, *n* (%)	399 (50.1%)
Age (years); mean ± SD	64.3 ± 13.3
**VTE, *n* (%)**	
PE alone	312 (39.2%)
DVT alone	292 (36.7%)
PE plus DVT	142 (17.9%)
Unusual site VTE	49 (6.2%)
PE, *n* (%)	454 (57.0%)
Central venous catheter, *n* (%)	217 (27.6%)
**Cancer type, *n* (%)**	
Colorectal	140 (17.6%)
Lung	116 (14.6%)
Breast	98 (12.3%)
Hematological	67 (8.4%)
Gynecological	53 (6.7%)
Bladder	44 (5.5%)
Prostate	41 (5.2%)
Pancreatic	35 (4.4%)
Kidney	35 (4.4%)
Brain	22 (2.8%)
ENT	30 (3.8%)
Others	107 (13.5%)
Metastases, *n* (%)	430 (55.5%)
Oncological treatment, *n* (%)	561 (70.9%)
ECOG >0 performance status, *n* (%)	481 (68.7%)
Anticoagulant treatment with LMWH	776 (97.7%)
Discontinuation anticoagulation	310 (38.9%)
**Complications**	
Death	488 (61.3%)
Recurrence VTE	95 (11.9%)
Clinically relevant bleeding	114 (14.3%)
Major bleeding	57 (7.2%)

SD, standard deviation; VTE, venous thromboembolism; PE, pulmonary embolism; DVT, deep thrombosis venous; ENT, ear, nose, and throat; ECOG, Eastern Cooperative Oncology Group; LMWH, low-molecular-weight heparin.

### 3.1. Characteristics of patients with incidental vs. symptomatic VTE

Those patients with incidental VTE had a higher prevalence of metastases (60.4% vs. 51.8%; OR: 1.4, 95% CI: 1.1–1.9; *p* = 0.018), PE (87.7% vs. 34%; OR: 13.8, 95% CI: 9.5–20.1; *p* < 0.0001), male sex (54.8% vs. 46.9%; OR: 1.2, 95% CI: 1.02–1.4; *p* = 0.021), and ECOG scores >0 (72.8% vs. 65.7%; OR: 1.4, 95% CI: 1.1–1.9; *p* = 0.045). The median duration of anticoagulant therapy was 9.5 (p25–p75, 6–17.8) and 9.7 (p25-p75, 4.8–20.8) months in patients with incidental and symptomatic VTE, respectively (*p* = 0.677). There were no differences in median follow-up in both groups (20.2 months [p25–p75, 8.9–37.6] vs. 20.8 months [p25–p75, 6.7–40.6]; *p* = 0.839). The characteristics of both groups are shown in Table 2.

**Table 2:** Comparison of baseline characteristics of patients with incidental and symptomatic VTE.

Variable	Incidental VTE (*n* = 341)	Symptomatic VTE (*n* = 455)	*p*-value
Male sex, *n* (%)	187 (54.8%)	214 (46.9%)	0.021
Age (years); mean ± SD	65.0 ± 13.1	63.8 ± 13.4	0.57
**VTE event, *n* (%)**			0.0001
PE	224 (65.7%)	88 (19.3%)	
DVT	11 (3.2%)	281 (61.9%)	
PE + DVT	75 (22.0%)	67 (14.8%)	
Unusual site VTE	31 (9.1%)	18 (4.0%)	
PE, *n* (%)	299 (87.7%)	155 (34.0%)	0.0001
Central venous catheter, *n* (%)	90 (26.5%)	128 (28.5%)	0.22
**Cancer type, n (%)**			0.0001
Colorectal	87 (25.5%)	53 (11.7%)	
Lung	63 (18.5%)	53 (11.7%)	
Breast	33 (9.7%)	65 (14.3%)	
Hematological	14 (4.1%)	54 (11.7%)	
Gynecological	21 (6.2%)	32 (7.1%)	
Bladder	20 (5.9%)	24 (5.3%)	
Prostate	8 (2.3%)	33 (7.3%)	
Pancreatic	17 (5.0%)	18 (4.0%)	
Kidney	13 (3.8%)	22 (4.9%)	
Brain	6 (1.8%)	16 (3.5%)	
ENT	18 (5.3%)	12 (2.6%)	
Others	41 (12.0%)	66 (14.6%)	
Metastases, *n* (%)	201 (60.4%)	229 (51.8%)	0.018
Oncological treatment, *n* (%)	235 (69.5%)	326 (72.0%)	0.45
ECOG >0 performance status, *n* (%)	219 (72.8%)	262 (65.7%)	0.045
Anticoagulant treatment with LMWH	337 (98.8%)	439 (96.9%)	0.072
Discontinuation of anticoagulation	146 (42.8%)	164 (36.0%)	0.053
**Complications**			
Death	224 (65.7%)	264 (58.0%)	0.028
Recurrence VTE	35 (10.3%)	60 (13.2%)	0.21
Clinically relevant bleeding	55 (16.1%)	59 (13.0%)	0.21
Major bleeding	26 (7.6%)	31 (6.8%)	0.66

SD, standard deviation; VTE, venous thromboembolism; PE, pulmonary embolism; DVT, deep thrombosis venous; ENT, ear, nose, and throat; ECOG, Eastern Cooperative Oncology Group; LMWH, low-molecular-weight heparin.

### 3.2. Primary outcome

Overall, 95 cases of recurrent VTE were noted during the follow-up period (11.9%; 95% CI: 9.8%–14.4%). Of these, 35 were incidental cases, while 60 were cases of symptomatic VTE. One-third (*n* = 32) occurred during anticoagulant treatment. Of note, 62.5% (20/32) had new metastases at the time of recurrent VTE event. The most frequent recurrent VTE included PE (40%) and lower limb DVT (37.8%). Additionally, the rate of recurrent VTE events in the incidental VTE group was 0.4 per 100 patients/month (95% CI: 0.3–0.5), while the rate in the symptomatic VTE group was 0.5 per 100 patients/month (95% CI: 0.4–0.6) (*p* = 0.313).

During follow-up, there were 114 CRB (14.3%; 95% CI: 12.0%–16.9%), of which 55 (48.2%) were in patients with incidental VTE. Of total, 12.3% were after stopping anticoagulant treatment, 75.4% were with low molecular weight heparin (LMWH), 7% with vitamin K antagonist and 5.3% with direct oral anticoagulants (DOACs). Previous to VTE, 14.9% (17/114) of patients had a CRB and the median time from CRB to VTE was 17.0 months (p25–p75, 4–25.9). Patients with incidental VTE had a lower prevalence of previous CRB (5.9% vs. 2.6%; OR: 0.43, 95% CI: 0.2–0.9; *p* = 0.027). The rate of CRB events in the incidental VTE group compared with that in the symptomatic VTE was 0.6 per 100 patients/month (95% CI: 0.5–0.8) vs. 0.5 per 100 patients/month (95% CI: 0.4–0.6), respectively (*p* = 0.128).

### 3.3. Secondary outcomes

**Figure 2:**
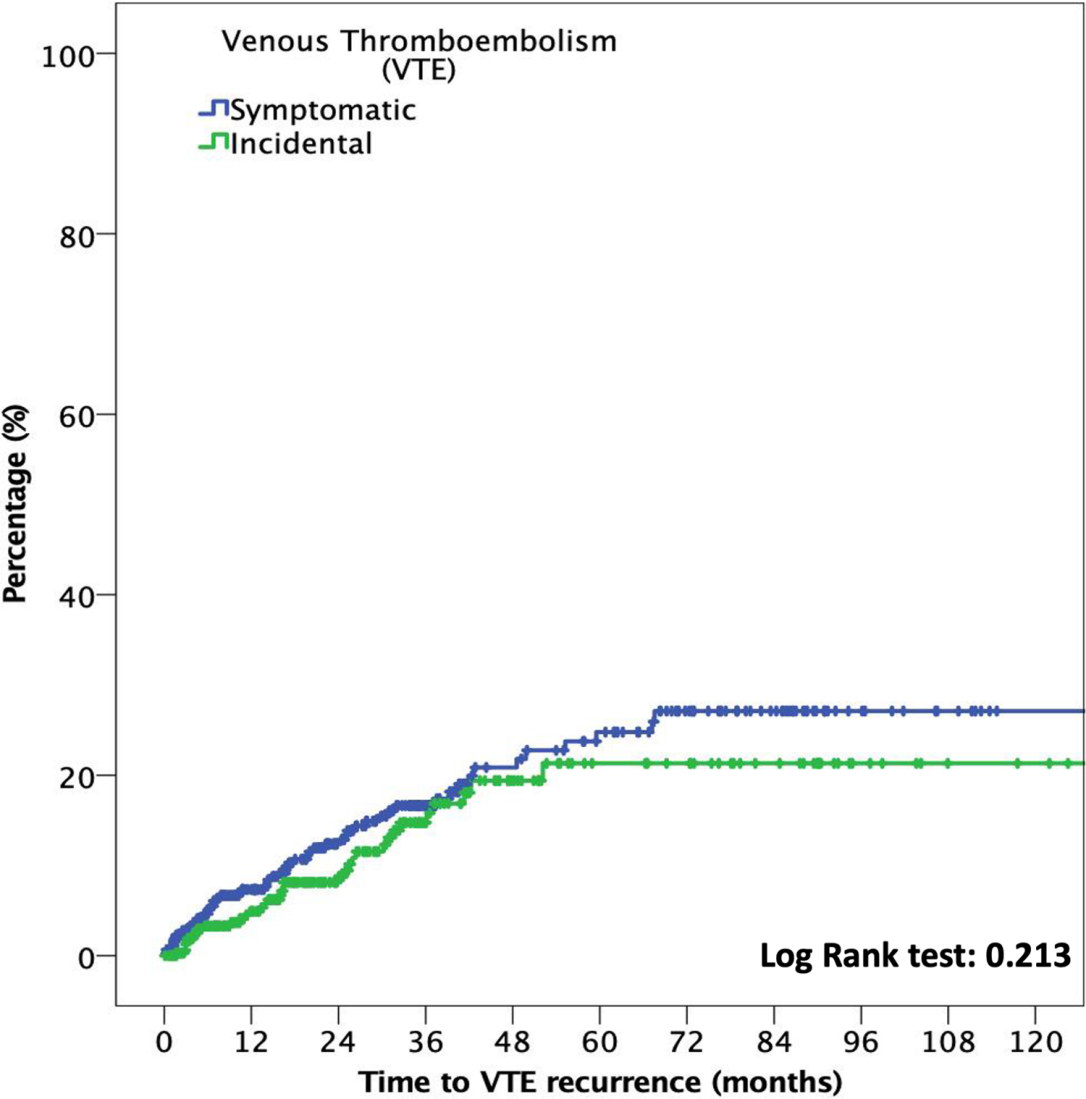
Cumulative recurrent VTE in patients with incidental and symptomatic VTE.

The median time to VTE was 18.3 months (p25–p75, 6.4–36.3). In patients with incidental VTE, the cumulative incidence of recurrent VTE at six months was 2.9% (95% CI: 1.4%–5.4%), compared with 4.2% (95% CI: 2.5%–6.5%) in patients with symptomatic VTE (OR: 0.7; 95% CI: 0.3–1.5; *p* = 0.372). At 12 months, the cumulative incidence of recurrent VTE was 4.1% (95% CI: 2.2%–6.9%) and 6.2% (95% CI: 4.1%–8.9%) in patients with incidental and symptomatic VTE, respectively (OR: 0.7; 95% CI: 0.4–1.3; *p* = 0.217). Figure 2 shows the time to VTE recurrence according to incidental or symptomatic VTE types.

**Figure 3:**
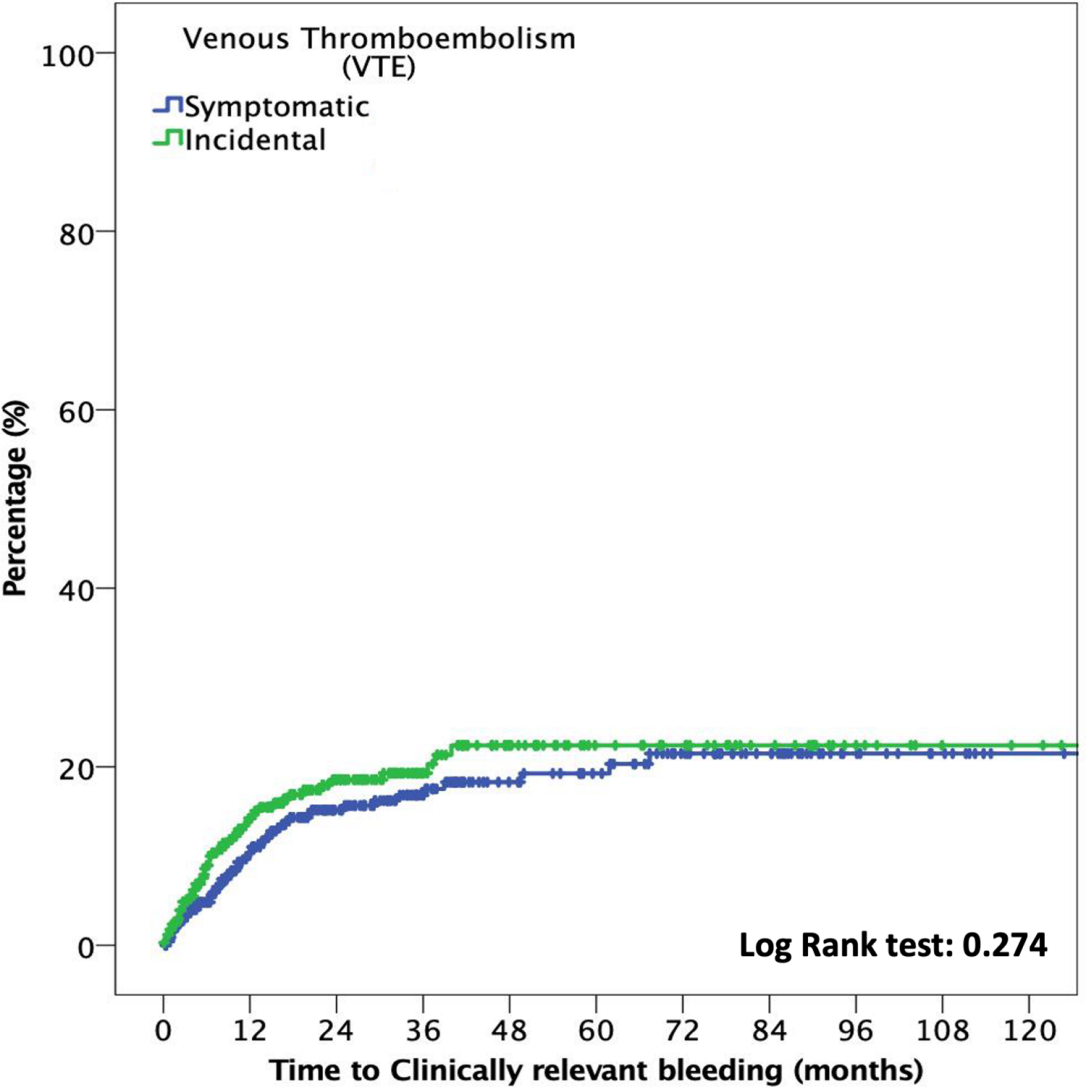
Cumulative clinically relevant bleeding in patients with incidental and symptomatic VTE.

Moreover, the median time to CRB was 16.8 months (p25–p75, 6.1–36.3). At six months, the cumulative incidence of CRB events in the incidental VTE group compared with that in the symptomatic VTE group was 7.9% (95% CI: 5.2%–11.5%) and 4.4% (95% CI: 2.7%–6.8%), respectively (OR: 1.8; 95% CI: 1.01–3.2; *p* = 0.046). No differences were noted in the cumulative incidence of CRB at 12-month follow-ups (12.3%; 95% CI: 8.9%–16.7% vs. 8.4%, 95% CI: 5.9%–11.5%; OR: 1.5; 95% CI: 0.95–2.3; *p* = 0.08)]. Figure 3 shows the time to CRB according to incidental or symptomatic VTE types.

Additionally, major bleeding occurred in 57 patients during follow-up (7.2%; 95% CI: 5.5%–9.1%), with a median time to major bleeding of 19.1 months (p25–p75, 6.8–37.6). The major bleeding rate was 0.3 per 100 patients/month (95% CI: 0.2–0.4) in patients with incidental VTE and 0.2 per 100 patients/month (95% CI: 0.2–0.3) in patients with symptomatic VTE (*p* = 0.502). The six-month cumulative major bleeding incidence was 3.8% (95% CI: 2.0%–6.5%) for patients with incidental VTE and 2.6% (95% CI: 1.4%–4.6%) for patients with symptomatic VTE (OR: 1.4; 95% CI: 0.7–3.2; *p* = 0.364). At 12 months, the cumulative incidence was 6.7% (95% CI: 4.3%–10.1%) and 4.4% (95% CI: 2.7%–6.8%) in patients with incidental and symptomatic VTE, respectively (OR: 1.5; 95% CI: 0.8–2.8; *p* = 0.164).

**Figure 4:**
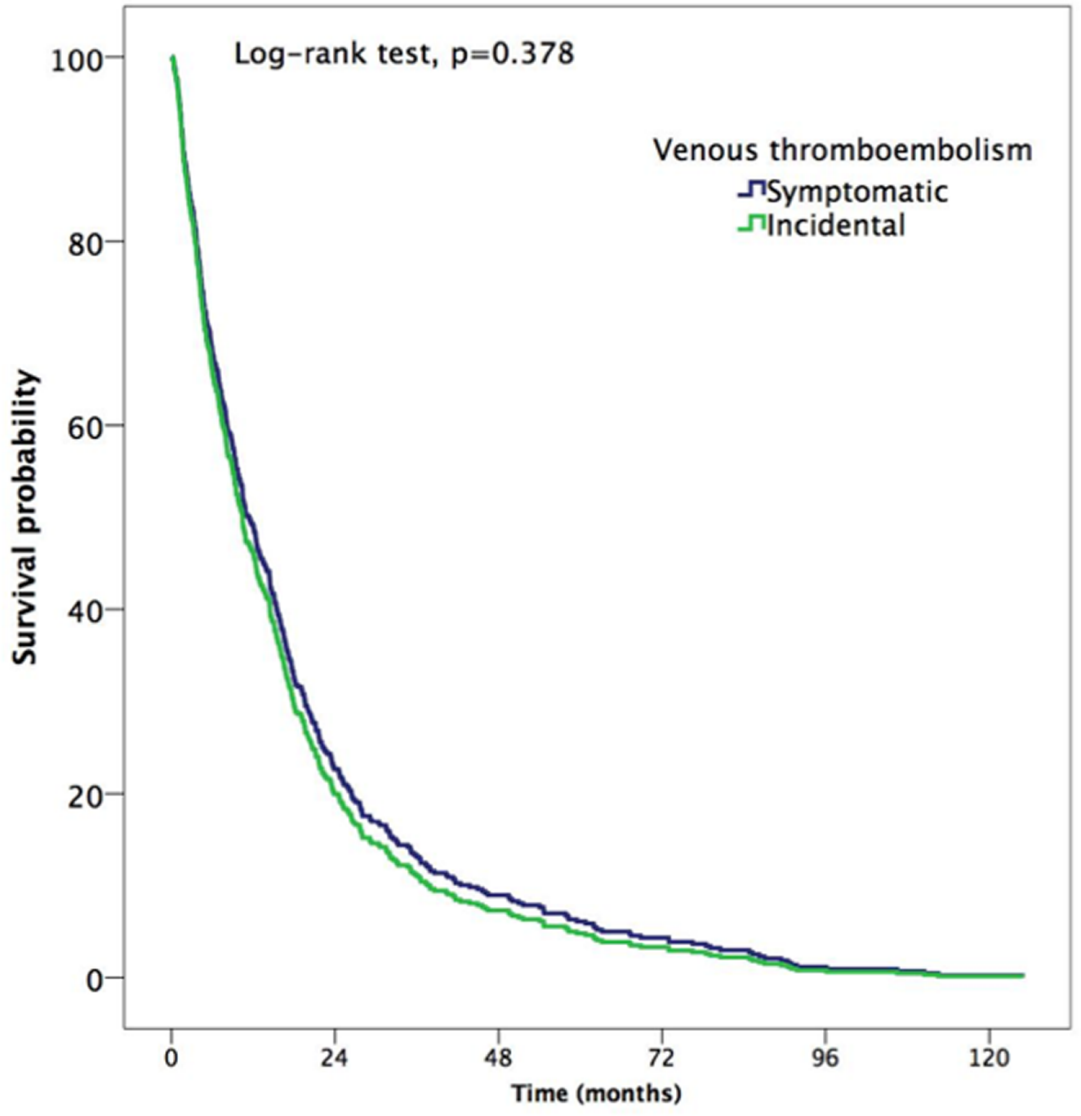
Survival of patients with incidental and symptomatic VTE; log-rank test, *p* = 0.378.

A total of 488 patients (61.3%; 95% CI: 57.8%–64.7%) died during follow-up, of which 32% (95% CI: 28.9%–35.3%) died at 12 months. 12 patients died of VTE and 2 of fatal bleeding. There were 224 (45.2%; 95% CI: 32.5%–58.3%) and 264 (38.4%; 95% CI: 30.3%–47.1%) deaths in patients with incidental and symptomatic VTE, respectively (HR: 1.1, CI: 0.9–1.4, *p* = 0.378). The median time to death was 10.7 months (p25–p75, 4.3–21.6) (Figure 4). At six months, the cumulative incidence of death in patients with incidental and symptomatic VTE was 18.5% (95% CI: 14.5%–23.7%) and 21.8% (95% CI: 17.7%–26.5%), respectively (OR: 0.8; 95% CI: 0.6–1.2; *p* = 0.311), while no differences were noted in the cumulative incidence of death at 12 months (31.7% [95% CI: 26.0%–38.2%] vs. 32.3% [95% CI: 27.3%–38.0%]; OR: 0.98; 95% CI: 0.8–1.2; *p* = 0.877).

In subgroup analyses of patients with incidental VTE, the variables associated with death included the presence of metastases (HRa: 2.2, 95% CI: 1.6–3.1, *p* < 0.001) and recurrent VTE (HRa: 1.7, 95% CI: 1.07–2.9, *p* = 0.026) (Table 3). Metastatic disease was associated with higher risks of recurrent VTE (HRa: 3.2, 95% CI: 1.4–7.5, *p* = 0.006) and CRB (HRa: 2.1; 95% CI: 1.0–4.1, *p* = 0.025, respectively) (Tables 4, 5). In subgroup analyses of those with symptomatic VTE, the variables associated with death included metastases (HRa: 2.1, 95% CI: 1.5–2.9, *p* < 0.001), ECOG scores >0 (HRa: 1.8, 95% CI: 1.3–1.5, *p* = 0.001), and recurrent VTE (HRa: 1.9, 95% CI: 1.2–2.9, *p* = 0.005) (Table 3). Moreover, the variable associated with recurrent VTE was metastasis (HRa 3.5, 95% CI: 1.9–6.4, *p* < 0.001) (Table 4), and the variable associated with CRB was male sex (HRa: 2.0, 95% CI: 1.1–3.7, *p* = 0.023) (Table 5).

**Table 3:** Cox regression with hazard ratios for overall mortality in cancer patients with incidental and symptomatic VTE.

Variable	HR (95% CI)	*p*-value	Adjusted HR (95% CI)	*p*-value
**Incidental VTE**				
Male sex	1.2 (0.9–1.7)	0.149	—	
Age (<65 vs. >65 years)	0.9 (0.7–1.2)	0.479	—	
Metastases	2.2 (1.5–3.2)	0.000	2.2 (1.6–3.1)	0.000
Oncological treatment	1.1 (0.8–1.6)	0.554	—	
ECOG performance status >0 (0 vs. >0)	1.4 (0.95–2.0)	0.094	—	
VTE recurrence	1.7 (1.02–2.8)	0.040	1.7 (1.07–2.9)	0.026
Clinically relevant bleeding	0.8 (0.6–1.2)	0.396	—	
**Symptomatic VTE**				
Male sex	1.2 (0.9–1.6)	0.131	—	
Age (<65 vs. >65 years)	1.2 (0.9–1.6)	0.253	—	
Metastases	2.0 (1.4–2.7)	0.000	2.1 (1.5–2.9)	0.000
Oncological treatment	1.3 (0.9–1.7)	0.123		
ECOG performance status >0 (0 vs. >0)	1.8 (1.3–2.5)	0.001	1.8 (1.3–2.5)	0.001
VTE recurrence	1.9 (1.2–2.9)	0.004	1.9 (1.2–2.9)	0.005
Clinically relevant bleeding	1.3 (0.9–1.6)	0.176	—	

HR, hazard ratio; CI, confidence interval; ECOG, Eastern Cooperative Oncology Group; VTE, venous thromboembolism.

**Table 4:** Cox regression with hazard ratios for recurrent VTE in cancer patients with incidental and symptomatic VTE.

Variable	HR (95% CI)	*p*-value	Adjusted HR (95% CI)	*p*-value
**Incidental VTE**				
Male sex	1.2 (0.6–2.5)	0.661	—	
Age (<65 vs. >65 years)	1.0 (0.5–2.1)	0.976	—	
Metastases	3.2 (1.4.0–7.9)	0.008	3.2 (1.4–7.5)	0.006
Oncological treatment	0.8 (0.3–2.0)	0.598	—	
ECOG performance status >0 (0 vs. >0)	0.6 (0.3–1.2)	0.155	—	
Clinically relevant bleeding	0.9 (0.3–2.6)	0.829	—	
**Symptomatic VTE**				
Sex (male)	1.2 (0.7–2.2)	0.543	—	
Age (<65 vs. >65 years)	1.4 (0.8–2.5)	0.247	—	
Metastases	3.7 (2.0–7.0)	0.000	3.5 (1.9–6.4)	0.000
Oncological treatment	1.5 (0.8–2.8)	0.199		
ECOG performance status >0 (0 vs. >0)	0.8 (0.5–1.5)	0.571	—	
Clinically relevant bleeding	0.6 (0.3–1.2)	0.170	—	

HR, hazard ratio; CI, confidence interval; ECOG, Eastern Cooperative Oncology Group; VTE, venous thromboembolism.

**Table 5:** Cox regression with hazard ratios for clinically relevant bleeding in cancer patients with incidental and symptomatic VTE.

Variable	HR (95% CI)	*p*-value	Adjusted HR (95% CI)	*p*-value
**Incidental VTE**				
Male sex	1.0 (0.6–1.8)	0.976	—	
Age (<65 vs. >65 years)	0.7 (0.4–1.4)	0.223	—	
Metastases	2.2 (1.1–4.4)	0.026	2.1 (1.0–4.1)	0.025
Oncological treatment	1.1 (0.6–2.3)	0.739	—	
ECOG performance status >0 (0 vs. >0)	1.1 (0.6–2.2)	0.761	—	
VTE recurrence	1.4 (0.5–4.0)	0.511		
**Symptomatic VTE**				
Male sex	2.1 (1.1–4.0)	0.016	2.0 (1.1–3.7)	0.023
Age (<65 vs. >65 years)	1.9 (1.03–3.4)	0.049	—	
Metastases	1.2 (0.6–2.3)	0.572	—	
Oncological treatment	0.7 (0.3–1.4)	0.243	—	
ECOG performance status >0 (0 vs. >0)	1.0 (0.6–2.0)	0.890	—	
VTE recurrence	0.7 (0.3–1.3)	0.307	—	

HR, hazard ratio; CI, confidence interval; ECOG, Eastern Cooperative Oncology Group; VTE, venous thromboembolism.

## 4. Discussion

This *post hoc* analysis from prospective studies of consecutive patients with cancer and VTE showed no differences in the rates of CRB and VTE recurrence between patients with incidental or symptomatic VTE in long-term follow-up periods. However, at six month follow-ups, patients with incidental VTE had more CRB (OR: 1.8; 95% CI: 1.01–3.2). Our findings support current recommendations regarding the management and treatment of these patients, which suggest that patients with incidental VTE receive the same anticoagulant therapy as those with symptomatic VTE (13–15).

The clinical characteristics of the patients included in this study were similar to those of other cohorts published (8, 11). For instance, the prevalence of metastases was significantly higher in patients with incidental VTE than in those with symptomatic VTE (60.4% vs. 51.8%; *p* = 0.02). The multicenter, observational, prospective EPIPHANY study in cancer patients, that included 604 patients with incidental PE and 429 patients with symptomatic PE, metastatic disease was more frequently observed in patients with incidental PE (78% vs. 68%, *p* = 0.004) (11). Additionally, in a prospective study of 165 patients with cancer and PE, Den Exter et al. reported that patients with incidental PE more frequently had metastases than those with symptomatic PE (69% vs. 67%, respectively) (8). In our study, patients with incidental VTE had worse performance statuses compared with patients with symptomatic VTE, indicated by ECOG scores >0 (72.8% vs. 65.7%, respectively; OR: 1.4 95% CI: 1.1–1.9; *p* = 0.04). In contrast, the EPIPHANY study found that patients with unsuspected PE had good performance statuses with ECOG scores between 0 and 1 (59% vs. 45%; *p* < 0.001) (11).

In our study, the incidence of CRB were 14.3% (95% CI: 12.0%–16.9%) during follow-up. In SELECT-D (multicenter, randomized and open-label study), the 6-month cumulative rate of clinically relevant nonmajor bleeding (CRNMB) was 4% (95% CI: 2%–9%) for dalteparin and 13% (95% CI: 9%–19%) for rivaroxaban (26). Caravaggio trial (open-label, multicentre study) showed that, at 6 months, major bleeding occurred 3.8% in the apixaban group and 4.0% in the dalteparin group (27). The TiCAT (Tinzaparin in Cancer-Associated Thrombosis) (21) and DALTECAN (Evaluation of Dalteparin for Long-term Treatment of Blood Clots in Subjects With Cancer) (28) study were prospective, one-arm studies that evaluated the safety of anticoagulant treatment beyond 6 months with tinzaparin and dalteparin, respectively. The TiCAT and DALTECAN study found a rate of major bleeding during 7–12 months was 2.1% and 2.4% in the period 7–12 months, respectively (27, 28). The incidence of HCR in our study is higher than that observed in clinical trials. This may be due to the fact that we included major bleeding and CRNMB. Another explanation is that our patients had a longer follow-up (median follow-up was 20.4 (p25–p75, 7.5–40.2).

At six month follow-ups, the cumulative incidence of CRB was significantly higher in patients with incidental VTE compared with in those with symptomatic VTE (7.9% vs. 4.4%, respectively; OR: 1.8; 95% CI: 1.01–3.2). These findings were consistent with a recent systematic review and meta-analysis of cancer patients with VTE (*n* = 12,977) (18). In this study, investigators reported a numerically higher risk of major bleeding events at six months in incidental VTE cases compared with symptomatic VTE cases (RR: 1.47, 95% CI: 0.99–2.20). One explanation of these findings is that patients with incidental VTE may have a higher prevalence of metastases compared with symptomatic VTE patients.

Or study showed that the incidence of recurrent VTE during the follow-up period was 11.9% (95% CI: 9.8%–14.4%). Investigators of the SELECT-D study (26) published a *post hoc* study in 2020. After 6 months of treatment for VTE with rivaroxaban or dalteparin, patients with residual deep vein thrombosis or pulmonary embolism were randomly assigned to 6 months of rivaroxaban or placebo (29). The cumulative VTE recurrence after 6 months was 14% in the placebo arm and 4% in the rivaroxaban arm (HR: 0.32; 95% CI: 0.06–1.58) (29). A *post hoc* study from the HOKUSAI-VTE Cancer trial (30) (open-label, multicentre, international) compared edoxaban and dalteparin beyond 6 months in cancer patients with VTE. Between 6 and 12 months, the incidence of recurrent VTE was 2.9% in dalterparin group and 1.4% in edoxaban group (31). A recent systematic review that included 11 studies showed that the rate of recurrent VTE beyond 6 months was variable, between 1%–12% (32). The high recurrence reported in our study might be explained by the high prevalence of patients with metastases and receiving cancer treatment.

In contrast, at six month follow ups, the cumulative incidence of recurrent VTE was numerically lower among patients with incidental VTE compared with those with symptomatic VTE (2.9% vs. 4.2%, respectively; OR: 0.7; 95% CI: 0.3–1.5). These results supported those observed in the study by Caiano et al., which reported a significantly lower rate of VTE recurrence at six months in patients with incidental VTE (RR: 0.62, 95% CI: 0.44–0.87) (18). The reasons for these findings are not clear. One of the hypotheses is that incidental VTE usually presents as subacute or chronic at the time of diagnosis, so could lead to a lower risk of recurrence. One international prospective study of 695 cancer patients with incidental PE reported 12-month recurrences of VTE and major bleeding rates of 6.0% (95% CI: 4.4%–8.1%) and 5.7% (95% CI: 4.1%–7.7%), respectively (17). Moreover, several retrospective studies have compared the rates of recurrence, major bleeding, and mortality between patients with clinically suspected and incidental PE. For instance, a cohort of 77 patients with lung cancer and PE (32 with incidental PE) reported similar rates of VTE recurrence in patients with incidental and suspected PE (19% vs. 20%, *p* = 1) (33). Similarly, Exter et al. did not find differences between patients with incidental and clinical PE in recurrence rates (13.3 vs. 16.9%; *p* = 0.77), major bleeding (12.5 vs. 8.6%; *p* = 0.5), or mortality (52.9 vs. 53.3%; *p* = 0.7) at 12-month follow-ups (8).

The EPIPHANY study also found similar rates of VTE recurrence and major bleeding in patients with incidental and symptomatic VTE, but with a lower 30-day mortality rate in patients with incidental PE (3%) compared with those with symptomatic PE (20%) (*p* < 0.0001) (11). A recent retrospective study that used data from the Registro Informatizado de la Enfermedad TromboEmbólica (RIETE) reported that cancer patients with incidental PE had a lower all-cause mortality rate than those with suspected PE (11% vs. 22%; OR: 0.43, 95% CI: 0.34–0.54) (16). However, no significant differences in PE recurrences (OR: 0.62, 95% CI: 0.25–1.54) or major bleeding (OR: 0.78, 95% CI: 0.51–1.18) were noted (16). Similarly, the first results of the multicenter, prospective TESEO study, that included 939 cancer patients with PE, showed a higher six-month survival in patients with incidental PE compared with those with suspected PE (80.9% vs. 55.5%; *p* < 0.0001) (34). However, the six-month mortality rate in patients with suspected PE was higher than that in other cohorts.

Our study also showed that patients with incidental or symptomatic VTE who had metastases were at a greater risk of mortality (HRa: 2.2, 95% CI: 1.6–3.1, *p* < 0.001 and HRa: 2.1, 95% CI: 1.5–2.9, respectively; *p* < 0.001). The presence of metastases has previously been shown to be associated with death. In the EPIPHANY study, for instance, metastatic disease was associated with death at the 30-day follow-up on multivariable analysis (HRa: 2.8, 95% CI: 1.4–5.5, *p* = 0.004) (11). However, this study analyzed the potential predictors of 30-day mortality in all cancer patients with PE, without comparing whether the PE was incidental or symptomatic. Our findings indicated that in both patient groups, VTE recurrence was associated with death (HRa: 1.7, 95% CI: 1.07–2.9, *p* = 0.026 and HRa: 1.9, 95% CI: 1.2–2.9, respectively; *p* = 0.005).

This study had several strengths. First, we recruited patients prospectively and consecutively as well as compared two cohorts (incidental vs. symptomatic VTE) during the same, long-term period. Currently, only a few studies have compared the outcomes between cancer patients with incidental vs. symptomatic VTE, and most of these studies have been retrospective and with small sample sizes and limited follow-up periods. Second, the sample size of our study was sufficient to perform subgroup analyses and identify the variables associated with death.

However, this study also had some limitations. First, this was a post-hoc study, and was therefore not initially designed to compare long-term complications in cancer patients with VTE. There was no control arm to compare results, although this aspect was solved, in part, with the comparisons between the two cohorts (incidental vs. symptomatic VTE). Third, we included all locations of venous thromboembolism (i.e., PE, DVT, and unusual locations), which may have created heterogeneity in the results, even though this aspect may have increased the study's representativeness of daily clinical practice. The inclusion of a high percentage of patients with metastases may have also biased the results due to high mortality rates. However, the data were similar to other cohorts, which we were able to adjust in order to perform subgroup analyses. Future studies may be needed to assess which patients within the incidental VTE group may require limited durations or amounts of anticoagulation therapy.

## 5. Conclusions

Cancer patients with incidental VTE and symptomatic VTE had similar rates of CRB and VTE recurrence in long-term follow-up. At six-month follow-up, CRB was significantly higher and VTE recurrence rates were lower in the incidental VTE group.

## Data Availability

The original contributions presented in the study are included in the article/Supplementary Material, further inquiries can be directed to the corresponding author.
