# Supplementary figures and images for: Comparison of long-term complications in cancer patients with incidental and acute symptomatic venous thromboembolism
Source: Front Cardiovasc Med. 2023 May 19;10:1118385. doi: 10.3389/fcvm.2023.1118385 (PMC10237269; doi:10.3389/fcvm.2023.1118385)

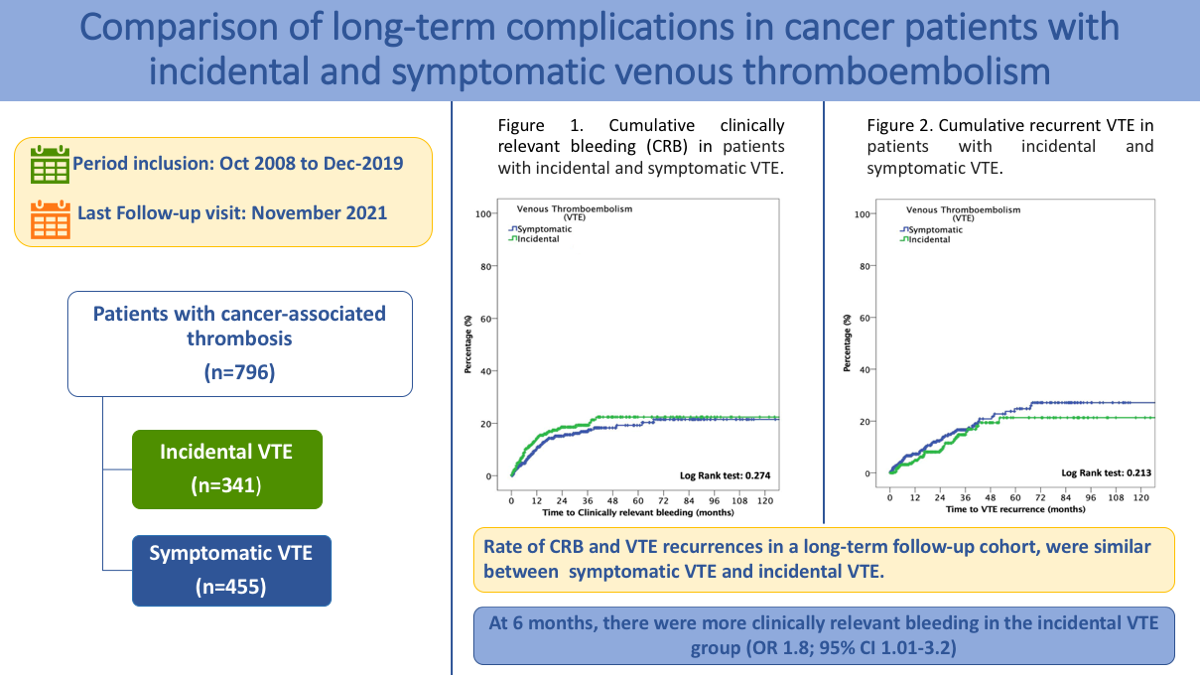

Supplement: Supplementary file 1 [file Image1.tiff]
